# Targeted autophagy: research progress on the regulatory potential of periodontitis prevention and treatment

**DOI:** 10.3389/fcell.2026.1708337

**Published:** 2026-04-21

**Authors:** Yujia Wang, Zhaojiang Fu, Yingtao Wu

**Affiliations:** Qingdao Stomatological Hospital Affiliated to Qingdao University, Qingdao, China

**Keywords:** autophagosome, autophagy, chronic periodontitis, mTOR, Porphyromonas gingivalis

## Abstract

Chronic periodontitis is a multifactorial inflammatory disease primarily caused by bacterial pathogens, leading to progressive destruction of both soft and hard periodontal tissues. While dental plaque biofilm has been universally recognized as the primary etiological factor in periodontitis development, disease progression is also influenced by local irritants and systemic modulators, making its pathogenesis complex and widely studied. Autophagy, an essential cellular self-protection mechanism, plays a pivotal role in maintaining intracellular homeostasis, responding to stress stimuli, and defending against microbial invasion. Recently, this process has emerged as a key research focus in periodontal pathology. This study investigates the dynamic changes and functional variations of autophagy during different stages of periodontitis, aiming to elucidate its precise role in disease initiation and progression.

## 1 Introduction

Chronic periodontitis is a chronic progressive inflammatory disease of periodontal tissues induced by bacterial pathogens. Its primary clinical manifestations include periodontal pocket formation and varying degrees of alveolar bone loss. In advanced stages, the condition can lead to tooth mobility, displacement, and even tooth loss (Kumar, 2019; Natto et al., 2018). Its occurrence is caused by bacteria, toxin factors, and various factors in the body. Autophagy refers to the process in which cells use lysosomes to degrade their own cytoplasmic proteins and damaged organelles under the regulation of autophagy related genes, playing a crucial role in maintaining cellular homeostasis, regulating cell growth and many other aspects. In recent years, studies have found a complex relationship between autophagy and the occurrence and development of periodontitis. This study aims to explore the role of autophagy in the occurrence and development of periodontitis, providing more evidence for targeted therapy of periodontitis.

### 1.1 The etiology and pathogenesis of periodontitis

Substantial experimental and clinical evidence has established periodontal disease as a microbially-induced infectious condition, with dental plaque biofilm serving as the initiating factor. This structurally complex microbial ecosystem adheres tenaciously to tooth surfaces, restorations, and gingival margins, creating an optimal environment for bacterial colonization, metabolism, and pathogenicity (Marsh, 2005). Bacteria rely on the unique structure of dental plaque biofilms to adhere to each other and grow in clusters, making it difficult to remove; The ability to resist surfactants and host defense functions can also be obtained through the formation process of biofilms (Dupont, 1997; Listgarten, 2000). These biofilm-specific properties fundamentally explain why dental plaque is universally recognized as the direct cause of periodontal diseases (Ramesh et al., 2016; Shibly et al., 2000; Lee et al., 2019).

From a pathophysiological perspective, the progression of periodontitis exhibits a gradual and dynamic process. The accumulation of plaque biofilm on tooth surfaces and within gingival sulci harbors pathogenic microorganisms that release substantial amounts of toxins and enzymes, including lipopolysaccharides (LPS) and proteases. These substances directly irritate periodontal tissues and activate the host’s immune defense system (Gasmi et al., 2022; Xu et al., 2020). With persistent inflammation, inflammatory cell infiltration markedly increases, accompanied by the excessive release of various inflammatory mediators such as interleukin-1β (IL-1β) and tumor necrosis factor-α (TNF-α), ultimately leading to the destruction of periodontal connective tissues and alveolar bone resorption (Meyle and Chapple, 2000; Curtis et al., 2000). This process involves the participation of multiple biological mechanisms within the organism. Autophagy, a highly conserved metabolic process ubiquitous in eukaryotic cells, functions as an intracellular “scavenger”, plays a pivotal role in maintaining cellular homeostasis, responding to diverse stress stimuli, and regulating cell growth, development, and death, among other critical biological functions (Glick et al., 2010; Liu et al., 2023). In recent years, as research on the relationship between autophagy and the pathogenesis of periodontitis has advanced, accumulating evidence suggests a complex and intricate interplay between autophagy and periodontitis (Greabu et al., 2020).

### 1.2 The fundamental mechanisms of autophagy

Autophagy is a cellular process responsible for degrading and recycling proteins and organelles to maintain intracellular homeostasis. It not only participates in the defense response of periodontal tissue cells against bacterial infection but also plays a crucial role in the initiation, progression, and regulation of inflammation. Furthermore, autophagy exerts profound effects on the survival, apoptosis, and tissue repair and regeneration of periodontal cells (Klionsky et al., 2021; Kim WJ. et al., 2022). In-depth research into the relationship between autophagy and periodontitis not only enhances our understanding of the disease’s pathogenesis from a novel perspective but also provides a theoretical foundation and potential therapeutic targets for developing novel treatment strategies based on autophagy modulation.

The autophagy process primarily consists of several key stages: autophagy initiation, autophagosome formation, fusion of autophagosomes with lysosomes, and substrate degradation. Under basal conditions, the mammalian target of rapamycin (mTOR)remains in an activated state and suppresses autophagy by phosphorylating autophagy-related proteins (Atgs). However, when cells are exposed to stimuli such as nutrient deprivation, oxidative stress, or pathogen infection, mTOR activity is inhibited, leading to the assembly of the ULK1-Atg13-FIP200 initiation complex, which triggers autophagy. Subsequently, Beclin-1 associates with class III phosphatidylinositol 3-kinase (PI3K-III) and other components to form a complex that promotes autophagosome membrane nucleation and elongation. The expanding autophagosome membrane engulfs cellular substrates, ultimately forming a double-membrane autophagosome. Once formed, the autophagosome is transported along microtubules to fuse with a lysosome, generating an autolysosome. Within the autolysosome, acid hydrolases degrade the sequestered cargo into small molecules, which are then released back into the cytoplasm for cellular reuse (Parzych and Klionsky, 2014).

## 2 Role of autophagy in periodontitis

### 2.1 Inflammatory cell infiltration and the inflammatory response

During the pathogenesis of periodontitis, excessive inflammatory responses can lead to severe damage and destruction of periodontal tissues. Periodontal pathogens and their metabolites, such as LPS released by *Porphyromonas gingivalis*, act as potent inflammatory stimuli that activate various cells within periodontal tissues, including macrophages, fibroblasts, and epithelial cells. In the inflammatory microenvironment of periodontitis, autophagy modulates immune responses by regulating the expression of inflammatory cytokines.

Infection by *P. gingivalis* can lead to excessive expression of inflammatory cytokines, exacerbating inflammatory damage in periodontal tissues. Autophagy may exert immunomodulatory effects by either degrading components of inflammasomes or suppressing the activation of inflammatory signaling pathways, thereby reducing the production of pro-inflammatory cytokines.

A prime example is the Nod-like receptor protein 3 (NLRP3) inflammasome, which plays a central role in the inflammatory response of periodontitis. Upon activation, the NLRP3 inflammasome rapidly promotes the maturation and secretion of inflammatory cytokines such as IL-1β and IL-18. Remarkably, autophagosomes can specifically recognize and engulf the NLRP3 inflammasome, subsequently transporting it to lysosomes. Within lysosomes, the potent hydrolytic enzymes degrade the NLRP3 inflammasome, thereby suppressing the excessive production of IL-1β and IL-18 at their source. This process effectively mitigates inflammatory damage in periodontal tissues (Hashim et al., 2024; Isola et al., 2022). And in the study by Yewon Yun et al., it was found that upregulation of autophagy can inhibit the activation of NLRP3 inflammasome and the release of its products, including pro-inflammatory cytokine IL-1β (Yun et al., 2021). Similarly, in the experiment of mouse peritoneal macrophages, silencing the autophagy regulatory gene PHB1 inhibited mitochondrial autophagy, resulting in abnormal activation of NLRP3 inflammasome and inhibition of pro IL-18 degradation process. Ultimately, the content of mature IL-18 in the cell supernatant increased significantly; And when the autophagy inhibitor 3-MA is added to further block autophagy, this phenomenon becomes more significant. From a molecular mechanism perspective, autophagy can form autophagosomes by encapsulating pro IL-18, which fuse with lysosomes to degrade it. At the same time, autophagy can also clear signals related to activating caspase-1, reducing the conversion of pro IL-18 to mature IL-18 (Harris et al., 2011; Chen et al., 2023).

Furthermore, autophagy regulates inflammatory cytokine expression by influencing the mitogen-activated protein kinase (MAPK) signaling pathway. Under physiological conditions, the MAPK pathway maintains moderate activity to regulate cell growth, differentiation, and stress responses. However, during periodontitis, periodontal pathogens and their metabolites hyperactivate the MAPK pathway, leading to significant upregulation of inflammatory cytokines such as TNF-α and IL-1β. Autophagy attenuates this excessive activation by degrading key signaling molecules in the MAPK pathway, including phosphorylated p38 MAPK and extracellular signal-regulated kinase (ERK) (Luo S. et al., 2024). This degradation mechanism effectively suppresses MAPK overactivation and subsequently reduces inflammatory cytokine production. Supporting evidence comes from a study by Liang L et al., which demonstrated that endothelin-1-treated periodontal ligament stem cells exhibited markedly elevated levels of inflammatory cytokines (TNF-α, IL-1β, and IL-6). Notably, this effect was significantly mitigated by inhibitors targeting ERK1/2, JNK, or MAPKs, confirming the pivotal role of MAPKs in regulating these inflammatory mediators (Liang et al., 2014). Meanwhile, Yifan Cheng et al. found that baicalin can reduce the ratio of autophagy related proteins Beclin-1 and LC3B-II/LC3B-I. When combined with autophagy inhibitor 3-methyladenine, the levels of IL-6 and IL-1β further decreased, indicating that baicalin reduces inflammation in periodontal ligament cells by inhibiting autophagy (Cheng et al., 2025)^.^

### 2.2 Promotion of inflammation resolution

Autophagy plays a crucial role not only in suppressing the initiation of inflammatory responses but also in facilitating the resolution of inflammation. During inflammatory processes, cells accumulate substantial amounts of damaged organelles (e.g., mitochondria, endoplasmic reticulum) and inflammatory mediators. These components persistently stimulate cells, leading to amplification of inflammatory signaling. Autophagy can sensitively recognize and encapsulate damaged organelles and inflammatory mediators through autophagosomes, fuse with lysosomes for degradation, thereby reducing the sustained stimulation of inflammatory signals and creating favorable conditions for timely resolution of inflammation and tissue repair (Zhou et al., 2024).

Autophagy exhibits sophisticated immunoregulatory capacity by modulating immune cell functions and enhancing anti-inflammatory cytokine production. A key mechanism involves facilitating macrophage polarization toward the anti-inflammatory M2 phenotype, which secretes potent anti-inflammatory mediators including interleukin-10 (IL-10), and researchers have clarified the association between autophagy mediated M2 polarization of macrophages and IL-10 secretion through experiments (Levin et al., 2016; Yang et al., 2025). IL-10 has a strong anti-inflammatory effect, which can inhibit the production of other inflammatory cytokines such as TNF-α and IL-1β, regulate the activity of immune cells, inhibit the infiltration of inflammatory cells, and further promote the resolution of inflammation (Fang et al., 2024). Jiang et al. found that autophagy activation of PDLSCs under mechanical stimulation can induce polarization of M1 macrophages by inhibiting the AKT signaling pathway, thereby facilitating bone reconstruction and tooth movement (Jiang et al., 2021).

### 2.3 Participate in antigen presentation

Autophagy also plays an important role in the process of antigen presentation. Dendritic cells (DCs) are the most powerful antigen-presenting cells in the body. Autophagy can promote the uptake and processing of pathogen associated antigens by DCs, presenting antigens to T cells through the major histocompatibility complex class II (MHC II) pathway and initiating adaptive immune responses. In periodontitis, the autophagy level of DCs may affect their ability to present periodontal pathogen antigens, thereby affecting the body’s immune response to periodontitis (Song et al., 2018). Sharawi H et al. characterized and detected gingival dendritic cells in 27 healthy subjects and 21 periodontal patients. The results showed that the frequency of Langerhans cells and plasma cell like dendritic cells in the gingiva of periodontitis patients was imbalanced, and the number of plasma cell like dendritic cells in diseased gingiva increased. The transformation of plasma cells like dendritic cells is accompanied by an increase in the expression of pro-inflammatory cytokines IL-1β, interferon (IFN)-α, and IFN-γ, while anti-inflammatory cytokine IL-10 is inhibited. This also indicates the role of dendritic cells in coordinating oral immunity and lays the foundation for evaluating and regulating immune changes associated with periodontitis (Sharawi et al., 2021). Also, a study demonstrated that the Mfa1 fimbriae of Porphyromonas gingivalis induce Bcl-2 expression via the Akt/mTOR pathway, which constitutes a key mechanism underlying the prolonged survival of dendritic cells. However, these abnormally surviving dendritic cells are impaired in efficient antigen presentation, thereby compromising the initiation of targeted immune responses and promoting the chronicity of inflammation (Meghil MM. et al., 2019).

## 3 The bone-immune response and alveolar bone resorption

Alveolar bone resorption is one of the important pathological changes in periodontitis. Promoting osteoblast differentiation to restore bone defects is particularly important in the treatment of periodontitis, and autophagy plays a positive regulatory role in osteoblast differentiation. Luo Z et al. demonstrated a novel autophagy activator CXM102, which can induce autophagy in bone marrow mesenchymal stem cells. The study found that CXM102 can activate bone marrow mesenchymal stem cells, promote the differentiation of osteoblast precursor cells into osteoblasts, and enhance the mineralization ability of osteoblasts (Luo Z. et al., 2024). In addition, autophagy is also involved in regulating the expression of osteogenic related genes. Research has found that autophagy related proteins such as Atg5 can interact with some transcription factors in osteoblasts, promoting the expression of osteogenic related genes such as Runx2 and osteocalcin (OCN). RUNX2 is a key transcription factor involved in osteoblast differentiation, regulating the proliferation of osteoblast mother cells and their differentiation into osteoblasts. Upregulation of its expression can promote the differentiation and maturation of osteoblasts (Vimalraj et al., 2015; Komori, 2019); OCN is an important component of bone matrix and a late stage marker of osteoblast differentiation. Its increased expression contributes to the synthesis and mineralization of bone matrix, and is considered an important indicator for evaluating cell differentiation into osteoblasts (Komori, 2020a; Komori, 2020b).

In normal periodontal tissue, osteoblasts and osteoclasts are in dynamic equilibrium, and autophagy not only affects osteoblasts, but also changes the level of autophagy proteins during osteoclast differentiation (Akisaka et al., 2001). Autophagic ablation can cause dysfunction of osteoblasts through endoplasmic reticulum stress and increase the secretion of RANKL (receptor activator of nuclear factor kappa B ligand), leading to activation of osteoclasts and bone resorption (Zhao et al., 2012). Studies have found that hypoxia enhances osteoclast differentiation and activity, as well as autophagy flow, by activating HIF-1 α (hypoxia inducible factor-1 α). Yi Zhao et al. found that HIF-1α induces the expression of its downstream target BNIP 3, increases the expression levels of autophagy related genes (such as Atg5 and Atg12), recruits LC3 to autophagosomes, and enhances the expression of RANKL, tissue protease K, etc., leading to an increase in osteoclastogenesis (Li et al., 2018). ShuJie Zhao et al. found that under lipopolysaccharide induced inflammatory conditions, miR-155 regulates osteoclast differentiation and activity by targeting TAB 2 (TGF β - activated kinase one binding protein 2), directly inducing osteoclast autophagy, which also confirms the regulatory effect of autophagy on osteoclasts (Zhao et al., 2018).

The key to periodontal pocket formation lies in the destruction of periodontal supporting tissues and the apical migration of epithelial attachment, a process in which autophagy is involved by regulating the secretion of matrix metalloproteinases (MMPs). Under inflammatory conditions, excessive autophagy can activate the MAPK/ERK signaling pathway in periodontal cells, promoting the expression and secretion of MMPs such as MMP-2, MMP-9, and MMP-13 (Beklen et al., 2007). These enzymes can degrade periodontal ligament collagen fibers, alveolar bone matrix, and basement membrane, leading to the destruction of periodontal connective tissue, loss of epithelial attachment, and ultimately the formation of periodontal pockets (Yang et al., 2021). In addition, autophagic dysregulation can cause an imbalance between proliferation and apoptosis of gingival epithelial cells, resulting in thickening and abnormal keratinization of the epithelial lining of periodontal pockets, which further aggravates the depth of periodontal pockets and the degree of inflammation (Jiang et al., 2020). Clinical studies have shown that treatment with SFD/CS/ZIF-8@QCT can demonstrate outstanding functions including antibacterial, immunomodulatory, osteogenic/angiogenic promotion, and recruitment promotion. Ultimately, it promotes good alveolar bone regeneration. At the same time, experiments indicated that the inhibition of osteogenesis/angiogenesis of PDLSCs by periodontitis is due to its strong suppression of energy metabolism and powerful activation of oxidative stress and autophagy. The synergistic effect of quercetin and zinc ions released from SFD/CS/ZIF-8@QCT can effectively reverse these biological processes (Yang et al., 2024).

## 4 The interaction between autophagy and periodontal pathogens

After periodontal pathogens such as *P. gingivalis* and *Actinobacteria* actinomycetes infect host cells, the body quickly responds and immune cells activate the defense system, one of which is the activation of autophagy. The initiation of autophagy relies on a series of complex signaling processes. Intracellular receptors can recognize pathogen related molecules, such as LPS on the surface of *P. gingivalis*. These pathogen related molecules bind to pattern recognition receptors (PRRs) on the cell surface, activating downstream signaling pathways and ultimately inducing the expression of autophagy related genes, promoting the formation of autophagosomes. Meghil MM. et al. (2019) found that the pili of *Pseudomonas* gingivalis can induce Akt nuclear localization and activation of dendritic cells derived from human monocytes, ultimately inducing mTOR. The activation of Akt/mTOR axis downregulates LC3 II in cells, which is also essential for autophagosome formation and maturation. The use of allosteric panAkt inhibitor MK2206 and mTOR inhibitor rapamycin confirmed the role of the Akt/mTOR signaling pathway in inhibiting dendritic cell autophagy in *P. gingivalis*.

Autophagy related proteins Atg5, Atg7, etc., play an important role in the clearance of *P. gingivalis* by cells. Cells lacking Atg5 or Atg7 have a significantly reduced ability to clear *P. gingivalis*, leading to a massive proliferation of bacteria within the cells and exacerbating cell damage. When autophagy is activated, Atg12 covalently binds to Atg5 and interacts with Atg16L1 to form a complex. This complex is located at the starting site of the autophagosome membrane and can promote membrane extension. Simultaneously forming LC3 III and specifically binding to the autophagosome membrane, labeling autophagosomes and promoting their maturation. During this process, autophagosomes gradually extend and precisely encapsulate invading periodontal pathogens, forming a complete autophagosome structure that separates bacteria from other cellular components (Fujioka and Noda, 2025).

The autophagosome encapsulating periodontal pathogenic bacteria will subsequently fuse with lysosomes to form autolysosomes. In an acidic environment, hydrolytic enzymes in lysosomes are activated to comprehensively and thoroughly degrade periodontal pathogens in autolysosomes. Research has shown that in autophagic lysosomes, the cell wall, membrane, proteins, nucleic acids, and other substances inside *P. gingivalis* are gradually broken down by hydrolytic enzymes, ultimately degrading into small molecule substances such as amino acids, nucleotides, etc. These small molecule substances can be reabsorbed and utilized by cells, providing the necessary material basis for cell metabolism and functional maintenance (Kang et al., 2022). Kang S et al. found that the ubiquitination of RAB7A in macrophages stimulated by Porphyromonas gingivalis can inhibit the function of active RAB7A in promoting lysosomal transport dynamics and autophagosome-lysosome fusion, thereby exacerbating the development of periodontitis. Meanwhile, the ubiquitination of RAB7A is mediated by USP4, and upregulation of USP4 can alleviate periodontitis *in vivo* (Kang et al., 2025).

In addition, autophagy can further enhance the killing ability of cells against periodontal pathogens by regulating the expression of intracellular antimicrobial peptides. Antimicrobial peptides are a class of small molecule peptides with broad-spectrum antibacterial activity that play an important role in host immune defense. Autophagy can promote the transcription and expression of antimicrobial peptide genes by activating relevant signaling pathways. For example, Shipra Vaishnava et al. (2008) found that autophagy in intestinal epithelial cells can activate the nuclear factor kappa B (NF -κB) signaling pathway, promoting the expression of antimicrobial peptides. It may be because NF -κB enters the nucleus and binds to specific sequences in the promoter region of antimicrobial peptide genes, thereby promoting antimicrobial peptide expression. Defensins in periodontal cells can disrupt the cell membrane structure of bacteria, leading to the leakage of bacterial contents and ultimately achieving the goal of killing bacteria. Meanwhile, autophagy can also provide sufficient energy and raw materials for the synthesis of antimicrobial peptides by regulating intracellular metabolic processes, further enhancing the cell’s defense against periodontal pathogens (Dommisch et al., 2012).

However, some periodontal pathogens can also evolve mechanisms to evade autophagic clearance. *Porphyromonas gingivalis* can secrete gingival protease, degrade autophagy related proteins, inhibit autophagosome formation, and thus evade autophagy clearance (Shiheido-Watanabe et al., 2023). In addition, *P. gingivalis* can inhibit autophagy initiation by activating the mTOR signaling pathway, which is beneficial for bacterial survival and reproduction within cells. Kyulim Lee et al. (2018) used high-resolution three-dimensional transmission electron microscopy to investigate the presence of *P. gingivalis* in ER rich double membrane autophagic vesicles. Inhibiting autophagy with 3-methyladenine or ATG5 siRNA significantly reduced the activity of *P. gingivalis* in gingival epithelial cells, revealing a new mechanism by which *P. gingivalis* successfully establishes a replication niche and persistence in oral mucosa by utilizing host autophagy mechanisms to survive in gingival epithelial cells.

## 5 Periodontal stability and development

In periodontitis, autophagy dysfunction leads to an imbalance in periodontal tissue homeostasis. On one hand, the protective effect of autophagy on periodontal cells is weakened, apoptosis increases, periodontal ligament fibers degrade, and gum recession occurs; on the other hand, immune imbalance in bone and enhanced alveolar bone resorption reduce periodontal support, ultimately resulting in the loss of periodontal stability (such as tooth mobility and displacement) (Kim WJ. et al., 2022; Ren et al., 2024; Liu et al., 2017). Furthermore, autophagy dysfunction also inhibits the repair function of PDLSCs, decreases periodontal tissue regeneration, and further exacerbates instability.

Moderate activation of autophagy can promote the restoration of periodontal stability through multiple mechanisms: clearing pathogenic bacteria and inflammatory factors to reduce tissue damage; enhancing PDLSC proliferation, migration, and differentiation to facilitate repair of the periodontal ligament, gums, and alveolar bone; regulating the balance between osteogenesis and bone resorption to inhibit alveolar bone loss and restore periodontal support (Heitz-Mayfield et al., 2002; Herrera et al., 2022; Sanz et al., 2020). *P*. *gingivalis* exhibits a dose-dependent and time-dependent inhibitory effect on alveolar epithelial cells, and it also promotes autophagy and apoptosis of alveolar epithelial cells in a dose-dependent manner. At the same time, using the autophagy inhibitor chloroquine (CQ) to inhibit autophagy or silencing LC3 with siRNA can significantly reduce *P. gingivalis* -induced cell apoptosis and affect cell viability (Zhao et al., 2024).

## 6 Autophagy-related targets and research progress in the treatment of periodontitis

The goal of periodontitis treatment is to control inflammation, prevent further damage to periodontal tissue, and promote periodontal tissue regeneration (Albeshri and Greenstein, 2022; Yan et al., 2020; Lei et al., 2022). With the development of biomedical engineering and regenerative medicine, stem cell therapy has received widespread attention and research aimed at improving the predictability and effectiveness of periodontal tissue regeneration. PDLSCs can differentiate into osteoblasts, fibroblasts, etc. (Xu et al., 2024; Bullon et al., 2012; Yu et al., 2024). Compared to other bone marrow mesenchymal stem cells, PDLSCs have stronger immune regulatory abilities and can suppress excessive inflammatory reactions by secreting TGF-β, PGE2, and other factors (Liu et al., 2019; Tomokiyo et al., 2019).

With the development of medicine, gene therapy has entered our field of vision. Gene therapy aims to treat diseases by introducing exogenous genes to correct or compensate for defective genes. Tsuchiya et al. (Tsuchiya et al., 2017) used *in vitro* electroporation to transfect the plasmid containing mouse BMP-4 cDNA (pCAGGS-BMP4) into cultured rat PDLSCs. Mature BMP-4 was produced and secreted in the cells, confirming that this method can be used for gene therapy targeting periodontal tissue and regulating alveolar bone remodeling. Although gene therapy is still in the research stage in the treatment of periodontitis, it also provides new ideas and directions for future treatments.

mTOR is an important pathway related to autophagy. Rapamycin, as a classic mTOR inhibitor, can activate autophagy in periodontal ligament stem cells in animal models, inhibit inflammatory cytokines, delay cell senescence, enhance osteogenic differentiation ability, reduce RANKL-induced bone resorption, and improve periodontal attachment levels (Joshi et al., 2025). Preclinical studies have shown that metformin can reduce the effects of LPS, improve LPS-inhibited autophagy pathways, and downregulate mTOR expression. In addition, metformin can also mitigate ligature-induced alveolar bone resorption, providing new evidence that metformin is a potential drug for treating periodontitis and a potential target for periodontal intervention (Sun et al., 2021).

Research has found that endoplasmic reticulum stress can mediate inflammation and the upregulation of MMPs by activating the p38 MAPK pathway, promoting bone loss through coordinating inflammatory responses, apoptosis, and autophagy. ERK inhibitors can block excessive autophagy, reduce the expression of MMP 2/9/13, and delay the formation of periodontal pockets (Yin et al., 2025). The PI3K/Akt pathway is involved in the survival and migration of periodontal cells; activating Akt can inhibit pathological autophagy and alleviate gingival epithelial cell apoptosis and attachment loss (Yang et al., 2023).

## 7 Discussion

**FIGURE 1:**
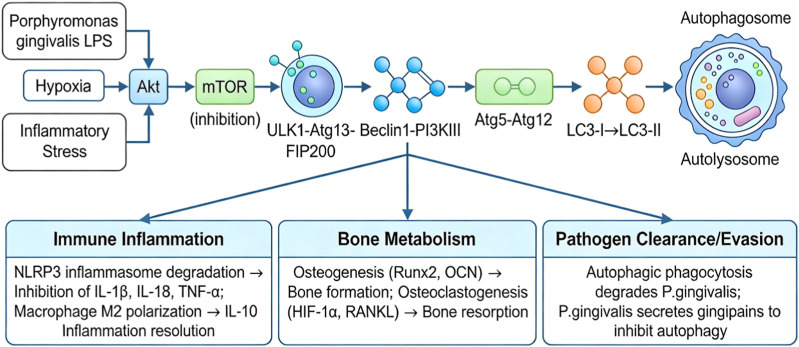
Molecular mechanisms of autophagy regulation in periodontitis.

In summary, autophagy plays multiple important roles in the occurrence and development of periodontitis, including the clearance of periodontal pathogens, regulation of inflammatory responses, and regulation of bone metabolism (Figure 1). The study of autophagy related signaling pathways provides a new perspective for a deeper understanding of the pathogenesis of periodontitis, as well as potential targets and new strategies for the treatment of periodontitis. However, there are still many problems and challenges in the current research on autophagy in periodontitis, such as the specific mechanism of autophagy in different periodontal tissue cells, the relationship between autophagy and other cellular biological processes, the association between periodontitis and other diseases, and how to achieve effective treatment of periodontitis by regulating autophagy. Future research needs to further explore the mechanism of autophagy in chronic periodontitis, develop more effective autophagy target regulating drugs, and provide a more solid theoretical basis and technical support for the clinical treatment of periodontitis.
